# New modification of the Perkow reaction: halocarboxylate anions as leaving groups in 3-acyloxyquinoline-2,4(1*H*,3*H*)-dione compounds

**DOI:** 10.1186/1860-5397-1-17

**Published:** 2005-12-09

**Authors:** Oldřich Paleta, Karel Pomeisl, Stanislav Kafka, Antonín Klásek, Vladislav Kubelka

**Affiliations:** 1 Department of Organic Chemistry, Prague Institute of Chemical Technology, Technická 5, 16628 Prague 6, Czech Republic; 2 Department of Chemistry and Enviromental Technology, Tomáš Bat'a University in Zlín, Nám. TGM 275, 762 72 Zlín, Czech Republic; 3 Zentiva, U Kabelovny 130, 10237 Prague 10, Czech Republic

## Abstract

Substituted 3-(fluoroacyloxy)quinoline-2,4(1*H*,3*H*)-diones including 3-(fluoroiodoacetoxy) derivatives react with triethyl phosphite to afford either the product of the Perkow reaction or the corresponding 4-ethoxyquinolin-2(1*H*)-one. In both reactions, the fluorocarboxylate anion acts as the first observed leaving group. This observation restricts the application of the intramolecular Horner-Wadsworth-Emmons synthesis to modify quinoline-2,4(1*H*,3*H*)-diones by the annulation of fluorinated but-2-enolide rings.

The 3-alkyl-3-hydroxyquinoline-2,4(1*H*,3*H*)-dione metabolites of *Pseudomonas* species exhibit antibiotic or lipoxygenase inhibitor activity[1] that may be modified by the introduction of an annulated pharmacophoric but-2-enolide ring. [2] The annulation was successfully carried out[3] (product **2**) via the intramolecular Wittig synthesis using a bromoacetyl derivative of the starting 3-hydroxyquinolinediones (**1**, Scheme 1).

**Scheme 1:**
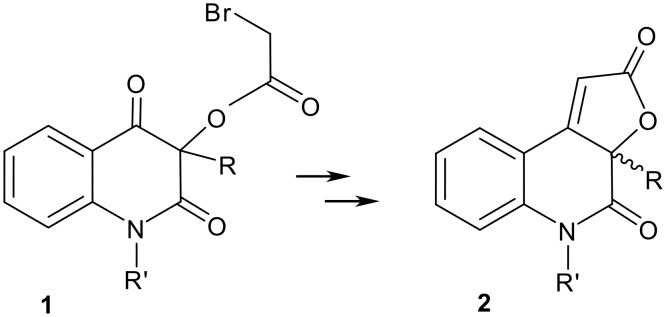
Annulation of the but-2-enolide ring.

Unlike haloalkanes and halocycloalkanes that undergo nucleophilic substitution reaction with trialkyl phosphites to afford the corresponding phosphonates according to the Michaelis-Arbuzov reaction,[4] the α-halocarbonyl compounds react with triethylphosphite to form enol phosphites **3** as a product of the Perkow reaction (Scheme 2). [5] The reaction is otherwise known to take place in halogenated ketones, diketones, aldehydes and also in some halogenoesters, amides or acyl halides. [5–6]

**Scheme 2:**
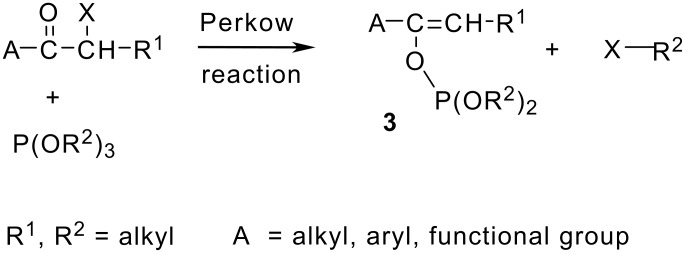
General Perkow reaction

Examples of the successful Perkow reaction also include chlorofluoro ketones, e.g. 1,3-dichloro-1,1,3,3-tetrafluoroacetone,[7] α-halonitroalkanes, and in rare cases also polycyclic compounds[8] such as 3,3-dichloro-1-methylquinoline-2,4(1*H*,3*H*)-dione. [9] In the Perkow reaction of halocompounds, the observed leaving groups were the halogen anions. However, in the reaction of pentaacetylated D-fructose with trimethyl phosphite the acetyloxy anion as the leaving group was also observed. [10] This rather unusual transformation may be attributed to the structural nature of the sugar skeleton and its multiple acetoxy groups.

In this communication we would like to report on the ability of halocarboxylate anions to act as leaving groups in the reactions of 3-(haloacyloxy)-quinoline-2,4(1*H*,3*H*)-diones with triethyl phosphite to afford **8** and **9**, the products of the Perkow reaction. Conversely, the 3-acetoxyquinoline-2,4(1*H*,3*H*)-diones bearing acetoxy group react via a different pathway.

The annulation resulting in the formation of the targeted product **10** was successfully carried out[3] by the intramolecular Wittig synthesis using the bromoacetyl derivative of the starting 3-hydroxyquinolinediones (Scheme 3, R^3^ = CH_2_Br, Y = H). In order to investigate the potential changes in biological activity[11–13] of **10**, we decided to introduce the fluorine substituent to the α-position of the butenolide ring (Scheme 3, **10**, Y = F) utilizing the fluorinated building-block approach for the annulation of the fluorobutenolide cycle. An analogous Horner-Wadsworth-Emmons synthesis using ethyl (diethoxyphosphoryl)-fluoroacetate prepared from ethyl fluoroiodoacetate has been reported for the preparation of the α-fluoro-α,β-unsaturated acyclic esters and lactones. [14] However, when 1-benzyl-3-butyl-3-(fluoroiodoacetoxy)quinoline-2,4(1*H*,3*H*)-dione (**4**) was reacted with triethyl phosphite an unexpected product **9** (Scheme 3) was obtained instead of the desired Horner-Wadsworth-Emmons intermediate 1-benzyl-3-butyl-3-(diethoxyphosphoryl)fluoroacetyloxy-quinoline-2,4(1*H*,3*H*)-dione. We assume the newly observed reaction to be due to the increased electron-withdrawing character of the acyl moiety.

**Scheme 3:**
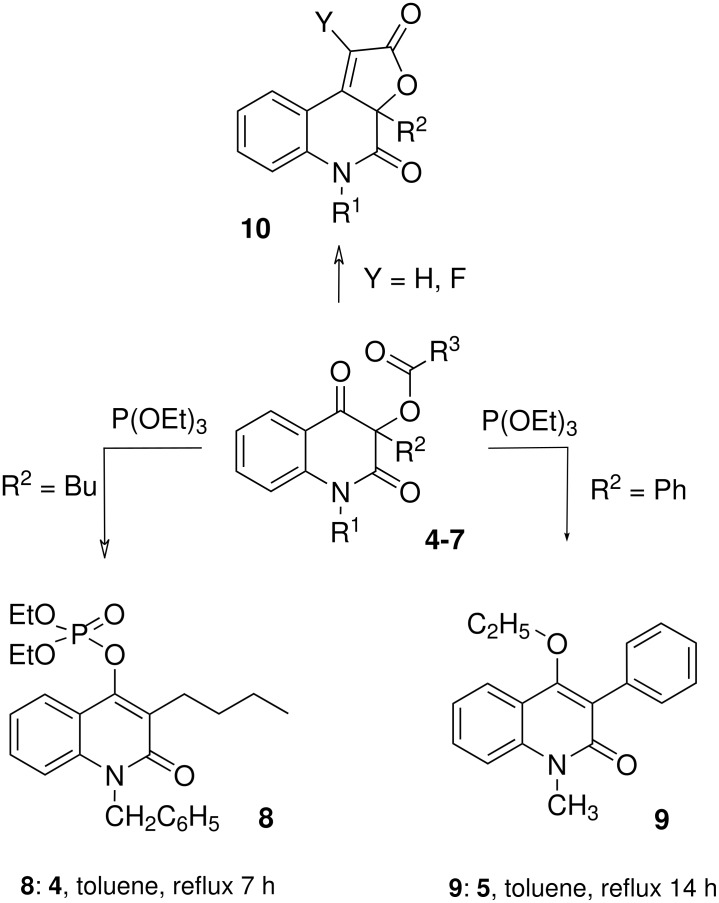
Novel reactions leading to the products **8** and **9**.

According to Scheme 2, the reaction of 3-(halogenoacyloxy)quinoline-2,4(1*H*,3*H*)-diones **4-6** with triethyl phosphite gave two kinds of products: the product of the Perkow reaction (**8**) and 1-alkyl-4-ethoxy-quinolin-2(1*H*)-one (**9**), respectively (Table 1, entries 3 and 5). In both the reactions, the halocarboxylate anion was eliminated. The role of halocarboxylates as leaving groups in the Perkow reaction is previously undescribed feature. Surprisingly, in contrast to the previously described Perkow reaction utilizing fructose pentaacetate,[10] the 3-acetoxyquinoline-2,4(1*H*,3*H*)-diones **7a** and **7b** did not react (Table 1, entries 6 and 7).

**Table 1:** Starting compounds **4–7** and the products **8** or **9** of their reactions with triethyl phosphite

Entry	Starting compound			Leaving group	Product	Yield (%)
		R^1^	R^2^			

1	**4**	PhCH_2_	Bu	CHFI-COO^(-)^	**8**	68
2	**5a**	PhCH_2_	Bu	C_3_F_7_O-CF(CF_3_)CF_2_O-CF(CF_3_)-COO^(-)^	**8**	18
3	**5b**	Me	Ph	C_3_F_7_O-CF(CF_3_)CF_2_O-CF(CF_3_)-COO^(-)^	**9**	11
4	**6a**	PhCH_2_	Bu	CF_3_COO^(-)^	**8**	36
5	**6b**	Me	Ph	CF_3_COO^(-)^	**9**	38
6	**7a**	PhCH_2_	Bu	[CH_3_COO^(-)^]	-	0
7	**7b**	Me	Ph	[CH_3_COO^(-)^]	-	0

In both of the reactions shown in Scheme 3 the elimination of the acyloxy moiety results in an energetically favored extension in conjugation of the π-system of the heterocyclic ring. In the reaction of the fluoroiodoacetyl derivative **4**, triethyl phosphite does not attack the C-I bond to form the corresponding product of Michaelis-Arbuzov reaction as is usual for the alkyl fluoroiodoacetates. [14] Instead, the phosphorus atom of triethyl phosphite attacks either the carbonyl group of the R^3^-CO acyl function, or the carbonyl located at C-4 on the heterocyclic ring (Scheme 4). Fluorinated acyloxy derivatives **5** and **6** reacted in a similar fashion at the carbonyl carbon atom of the acyl group or at C-4 of the heterocyclic ring (Table 1). As the acetoxy derivatives **7a** or **7b** did not react at all (Table 1, entries 6 and 7) it can be inferred that the new reactions (Scheme 2) require the presence of a stronger electron-withdrawing acyl group. Halogenated acyloxy groups possess a more reactive carbonyl group and as such are better leaving groups. Perhaps the increase in reactivity of the C4-carbonyl group is also due to halogenated acyloxy group. In any case, in the compounds **4–6** the two carbonyl groups are preferential reaction sites for the attack by the phosphorus atom of triethyl phoshite (Scheme 4).

**Scheme 4:**
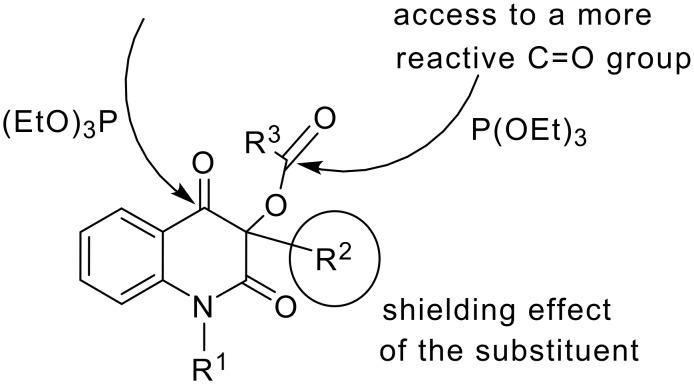
Reaction sites for the attack by P(OEt)_3_.

The factors directing the phosphite attack to form the enol phosphite **8**, the usual product of the Perkow reaction, or the unusual Perkow product **9** may be formulated as follows: As the Perkow reaction leading to the Perkow product **8** was observed for the compounds that all possess a butyl substituent at C-3 (**4**, **5a** and **6a**), a steric shielding effect is likely preventing the relatively bulky triethyl phosphite from attacking the acyloxy moiety. The nucleophilic attack then occurs at the ring C-4 carbonyl group. For this kind of Perkow reaction (Scheme 5), a hypothetical intermediate **11** and mechanism featuring acyloxy leaving group may be drawn. The proposed mechanisms in intermediates **11** and **12** featuring the acyloxy-leaving group corresponds to the proposed mechanisms[5,9,15–16] of the Perkow reaction (Scheme 5). The product **9** is most likely the result of an attack of the acyl-carbonyl moiety in the 3-acyloxyquinolinedione derivatives **5b** and **6b**. Both compounds possess a rigid phenyl substituent at C-3. In the case of **5b** and **6b**, the triethyl phosphite nucleophile attacks the more reactive carbonyl carbon in the ester group, because this reaction site is less shielded than in **4**, **5a** and **6a**. [17]

**Scheme 5:**
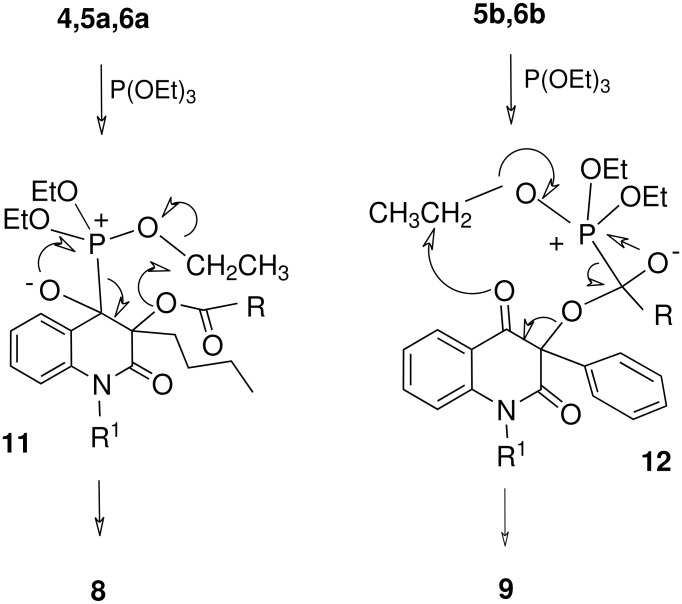
Hypothetic intermediates in the formation of the products **8** and **9**.

A hypothetical intermediate **12** and the proposed mechanism for formation of product **9** is depicted in Scheme 5. An analogous mechanism featuring fluoride anion as a leaving group was proposed previously for the reaction of perfluorinated aliphatic ketones with trialkyl phosphites. [7] The products **8** and **9** were obtained in isolated yields of 18–68% and 11–38% (Table 1), owing in part to the difficulties with their separation. In fact, the reactions reached an almost complete conversions of the starting acyloxy compounds **4–6** after 2–26 hours, and the isolated products **8** or **9** were the major or exclusive compounds in the reaction mixtures according to TLC analyses.

The reactions reported in this communication show that the application of the Horner-Wadworth-Emmons strategy for the annulation of the α-fluorobut-2-enolide ring to hetero(poly)cyclic systems using (diethoxyphosphoryl)fluoroacetyl intermediate may be difficult due to the competing reaction of trialkyl phosphite at highly reactive carbonyl group(s).

## Supporting Information

File 1 New modification of the Perkow reaction: halogenocarboxylate anions as leaving groups in 3-acyloxyquinoline-2,4(1*H*,3*H*)-dione compounds examples of the experimental procedures
