# Supplementary material for: New modification of the Perkow reaction: halocarboxylate anions as leaving groups in 3-acyloxyquinoline-2,4(1H,3H)-dione compounds
Source: Beilstein J Org Chem. 2005 Dec 9;1:17. doi: 10.1186/1860-5397-1-17 (PMC1399465; doi:10.1186/1860-5397-1-17)
Supplement: File 1 — New modification of the Perkow reaction: halogenocarboxylate anions as leaving groups in 3-acyloxyquinoline-2,4(1H,3H)-dione compounds examples of the experimental procedures [file Beilstein_J_Org_Chem-01-17-s001.doc]

### Supporting information:

**New Modification of the Perkow Reaction: Halocarboxylate Anions as Leaving Groups in 3-Acyloxyquinoline-2,4(1*H*,3*H*)-dione Compounds**

Oldřich Paleta,^a*^ Karel Pomeisl,^a^ Stanislav Kafka,^b^ Antonín Klásek,^b^ and Vladislav Kubelka^c^

## Typical procedures:

**[1-Benzyl-3-butylquinoline-2,4(1*H*,3*H*)-dione-3-yl]-(2-fluoro-2-iodoacetate) (4)**

Fluoroiodoacetyl chloride (329 mg, 1.48 mmol) was dropwise added to a mixture of 1-benzyl-3-butyl-3-hydroxyquinoline-2,4(1*H*,3*H*)-dione (300 mg; 0,93 mmol) in dry dichloromethane (5 mL) and pyridine (92 mg, 1.12 mmol) at -20°C while stirring and the mixture was then stirred at r.t. for 14 h. After removing the sovent, the residue was column chromatographed (neutral Al_2_O_3_, 30 g, benzene/EtOAc, 4:1). Product **2** as amorphous white solid evolving iodine, yield 227 mg (49 %).

^1^H NMR (CDCl_3_, 300.1 MHz) δ: 0.86 (t, C*H*_3_CH_2_, ^3^*J*_HH_=7.1 Hz, 3H), 1.29 (se, CH_3_C*H*_2_, ^3^*J*_HH_=7.3 Hz, 2H), 1.42 (qi, CH_2_C*H*_2_CH_2_, ^3^*J*_HH_=7.8 Hz, 2H); 1.97-2.23 (m, C*H*_2_-C-O, 2H), 5.32 (d, 1H, PhC*H*_a_H_b_, ^2^*J*_HaHb_=16.5 Hz ), 5.36 (d, PhCH_a_*H*_b_, ^2^*J*_HbHa_=16.5 Hz, 1H), 7.06 (dd, C-H Ar, ^3^*J*_HH_=8.2 Hz, ^4^*J*_HH_=2.2 Hz, 1H), 7.16 (dt, C-H Ar, ^3^*J*_HH_=7.7 Hz, ^4^*J*_HH_=2.2 Hz, 1H), 7.20-7.40 (m, C-H Ar, 5H), 7.37 (d, CHFI, ^2^*J*_HF_=51.1 Hz, 1H), 7.51 (m, C-H Ar, 1H) 8.0 (dd, C-H Ar, ^3^*J*_HH_=7.7 Hz, ^4^*J*_HH_=1.7 Hz, 1H). ^19^F NMR (CDCl_3_, 376.5 MHz) δ: -162.50 (d, CH*F*I, ^3^*J*_HF_=50.7 Hz, 1F). ^13^C NMR (CDCl_3_, 75.5 MHz, H decoupling) δ: 14.3 (s, CH_3_), 23.0 (s, CH_3_*C*H_2_), 25.3 (s, CH_3_CH_2_*C*H_2_), 36.9 (s, CH_3_CH_2_CH_2_*C*H_2_), 47.1 (s, Ph*C*H_2_), 56.4 (d, *C*HFI,^1^*J*_CF_=263.4 Hz), 86.4 (s, CHFICOO-*C*), 116.9, 124.5, 127.1, 128.4, 129.5, 129.8, 137.3 (7xs, C-H Ar), 121.0, 136.1, 142.6 (3x s, *C*=CH Ar), 165.9 (d, CHFI*C*OO, ^2^*J*_CF_=28.3 Hz), 169.0 (s, CON), 190.6 (s, C=O). EIMS *m/z* Calcd. for C_22_H_21_FINO_4_ 509.32; Found 509 (M^+^), 306 (52; M^+^-CHFICOO), 277 (11), 266 (15), 234 (5), 208 (7), 146 (5),103 (2), 91 (100), 77 (5), 67 (16).

**[1-Methyl-3-phenylquinoline-2,4(1*H*,3*H*)-dione-3-yl]-[2,5-bis(trifluoromethyl)-**

**2,4,4,5,7,7,8,8,9,9,9-undecafluoro-3,6-dioxanonanoate] (5b):**

A flask (25 mL) was charged with 3-butyl-1-methyl-3-hydroxy-quinoline-2,4(1*H*,3*H*)-dione (100 mg, 0.37 mmol) in dichloromethane (5 mL) and pyridine (29.3 mg; 0.37 mmol) and immersed in ice-bath. 2,5-Bis(trifluoromethyl)-2,4,4,5,7,7,8,8,9,9,9-undecafluoro-3,6-dioxanonanoyl fluoride (224 mg, 0.45 mmol) was then added dropwise through septum while stirring (magnetic spinbar). The mixture was allowed to warm to r.t. and stirred for 14 h. The solvent was removed on rotary evaporator and the residue subjected to column chromatography (silica gel, 10 g, benzene/EtOAc, 4:1). Ester **5b** as slightly yellow oil, yield 265 mg (95 %).

^1^H NMR (CDCl_3_, 300.1 MHz) δ: 3.57 (d, CH_3_-N, ^5^*J*_HH_= 4.4 Hz, 3H), 7.19 (m, C-H Ar, 2H), 7.40 (m, C-H Ar, 5H), 7.63 (t, C-H Ar, ^3^*J*_HH_= 7.7 Hz, 1H), 7.97 (t, C-H Ar, ^3^*J*_HH_= 7.7 Hz, 1H). ^19^F NMR (CDCl_3_, 376.5 MHz) δ, 2 diastereoisomers, A (50%, rel.), B (50% rel.): -79.7 (m, OCF(CF_3_)C*F*_2_, ^2^*J*_FF_=150.6 Hz, , 2F); -80.4 (m, C*F*_3_CF_2_CF_2_, 3F); -81.9 (m, OCF(C*F*_3_) CF_2_, 3F),

-82.4 (m, OCF(C*F*_3_)-COO, ^3^*J*_FF_=15.4 Hz, 3F); -82.9 (2x d, A, CF_3_CF_2_C*F*_2_, ^2^*J*_FaFb_= ^2^*J*_FbFa_=150.6 Hz, 2F); -83.6 (2xd, B, CF_3_CF_2_C*F*_2_, ^2^*J*_FaFb_=^2^*J*_FbFa_= 150.6 Hz, 2F); -130.0 (2xs, CF_3_C*F*_2_CF_2_, 3F),

-132.7 (dd, A, OC*F*(CF_3_)-COO, ^4^*J*_FF_= 20.3 Hz, 1F), 132.9 (m, B, OC*F*(CF_3_)-COO, 1F), -145.1 (t, A, OC*F*(CF_3_)CF_2_, ^3^*J*_FF_= 21.8 Hz, 1F), -145.5 (dd, B, OC*F*(CF_3_)CF_2_, ^3^*J*_FF_= 21.1 Hz, 1F). EIMS *m/z* Cald. for C_25_H_12_F_17_NO_6_ 745.34; Found 250 (M^+^ - C_3_F_7_CF(CF_3_)CF_2_OCF(CF_3_)COO, 31), 266 (100), 222 (23), 194 (3), 169 (C_3_F_7_^+^, 28), 152 (4), 125 (4), 105 (PhCO^+^, 85), 77 (28), 69 (CF_3_^+^, 32), 51 (CHF_2_^+^, 7).

**Reaction of 4 with triethyl phosphite:1-benzyl-3-butyl-4-[(diethoxyphosphoryl)oxy]**

**quinolin-2(1*H*)-one (8):**

A mixture of (quinolinedione-3-yl)-fluoroiodoacetate **4** (168 mg, 0.34 mmol), triethyl-phosphite (562 mg, 3.38 mmol) and toluene (5 mL) was refluxed for 7 h. After removing off toluene and triethyl phosphite, the crude product was chromatographed (neutral Al_2_O_3_, 30 g, benzene/EtOAc, 4:1) to obtain product as white crystals, yield 102 mg (68 %). For analysis, **9** was crystallised (acetone-hexane), yield 61 mg (41 %), m.p. 94-97 ^o^C.

IR (CHCl_3_) 3018 (m), 1642 (s), 1600 (m), 1052 (s) cm^-1^.^1^H NMR (CDCl_3_, 300.1 MHz) δ: 0.96 (t, C*H*_3_(CH_2_)_3_, ^3^*J*_HH_=7.1 Hz, 3H), 1.36 (dt, C*H*_3_CH_2_-OP, ^3^*J*_HH_=7.1 Hz, ^4^*J*_HP_=1.1 Hz, 6H), 1.45 (se, CH_3_C*H*_2_(CH_2_)_2_,^3^*J*_HH_= 7.4 Hz, 2H), 1.62 (qi, C*H*_2_CH_2_C=C, ^3^*J*_HH_=7.7 Hz, 2H), 2.85 (t, CH_2_C*H*_2_C=C, ^3^*J*_HH_= 8.0 Hz, 2H); 4.26 (m, CH_3_C*H*_2_-O, 4H), 5,55 (bs, PhC*H*_2_, 2H), 7.16-7.34 (m, C-H Ar, 7H), 7.41 (ddd, C-H Ar, ^3^*J*_HH_=7.1 Hz, ^4^*J*_HH_=1.7 Hz, 1H), 8.00 (dd, C-H Ar, ^3^*J*_HH_=8.0 Hz, ^4^*J*_HH_= 1.4 Hz, 1H). ^13^C NMR (CDCl_3_, 75.5 MHz, H decoupling) δ: 14.3 (s, *C*H_3_(CH_2_)_3_), 16.47, 16.54 (2×s, *C*H_3_-CH_2_-OP), 23.04, 26.0, 30.6 (3xs, C-*C*H_2_-C), 46.9 (s, Ph*C*H_2_), 65.5, 65.6 (2×s, CH_3_*C*H_2_-OP), 115.2, 122.7, 124.6, 127.2, 127.8, 129.4, 131.0 (7xs, C-H Ar), 124.6 (d, PO-C=C, ^3^*J*_CP_= 5.3 Hz), 117.9, 137.0, 138.5 (3xs, C-H Ar), 152.2 (d, PO-C=, ^2^*J*_CP_= 8.6 Hz), 164.0 (s, CON). ^31^P NMR (CDCl_3_, 202.5) δ: -5.19 (qi, ^3^*J*_PH_= 15.1 Hz, 1P). Calcd. for C_24_H_30_O_5_NP C, 65.00 % H, 6.81 % N, 3.16 %; Found C, 65.00 % H, 6.82 % N, 3.06 %.

**Reaction of 5b with triethyl phosphite:** **4-ethoxy-1-methyl-3-phenylquinolin-2(1*H*)-one (9):**

A mixture of quinolinedione **5b** (245 mg, 0.37 mmol) triethyl phosphite (1.91 g, 11.56 mmol) and toluene (5 mL) was refluxed for 14 h. Column chromatography (neutral Al_2_O_3_, 30 g, benzene/EtOAc, 5:1). Product **9** as white crystals, yield 11 mg (11 %), m.p. 98-101 °C. IR (CHCl_3_) 3010 (w), 1624 (s), 1592 (m) cm^-1^. ^1^H NMR (CDCl_3_, 300.1 MHz) δ: 1.16 (t, C*H*_3_CH_2_-O, ^3^*J*_HH_=7.1 Hz, 3H), 3.64 (q, CH_3_C*H*_2_-O, ^3^*J*_HH_=7.1 Hz, 2H), 3.75 (s, CH_3_-N, 3H), 7.24-7.55 (m, C-H Ar, 7H), 7.60 (m, C-H Ar, ^3^*J*_HH_= 7.2 Hz, ^4^*J*_HH_= 1.7 Hz, 1H); 8.03 (dd, C-H Ar, ^3^*J*_HH_= 8.0 Hz, ^4^*J*_HH_= 1.4 Hz, 1H). ^13^C NMR (CDCl_3_, 75.5 MHz, ^1^H decoupling) δ: 15.4 (s, *C*H_3_-CH_2_-O), 29.8 (s, CH_3_-N), 69.2 (s, CH_3_-*C*H_2_-O), 113.8, 121.8, 124.2, 127.6, 127.9, 130.6, 130.7 (7xs, C-H Ar), 119.7 (s, O-C=*C*), 118.7, 133.7, 139.3 (3xs, *C*=CH Ar), 159.6 (s, O-C=), 163.3 (s, CON). Calcd. for C_18_H_17_O_2_N C, 77.40 % H, 6.13 % N, 5.30%; Found: C, 77.19 % H, 6.20 % N, 5.01 %.

# Analytical data

**[1-Benzyl-3-butylquinoline-2,4(1*H*,3*H*)-dione-3-yl]-[2,5-bis(trifluoromethyl)-**

**2,4,4,5,7,7,8,8,9,9,9-undecafluoro-3,6-dioxanonanoate] (5a):**

Column chromatography (silica gel 10 g, benzene-EtOAc 4:1) afforded ester **5a** as colourless oil, yield 245 mg (95 %).

^1^H NMR (CDCl_3_) δ: 0.87 (t, 3H, , CH_3_, ^3^*J*_HH_=7,1 Hz); 1.29 (m, 2H, CH_3_-C*H*_2_-); 1.43 (m, 2H,CH_3_CH_2_C*H*_2_ ); 2.14 (m, 2H,-CH_2_-C-O) ; 5.30 (s, 2H, , Ph-CH_2_-); 7.07 (m, 1H, -CH=); 7.17 (t, 1H, -CH=, ^3^*J*_HH_= 7,7 Hz); 7.22-7.38 (m, 5 H, -CH=), 7.52 (t, 1H, -CH=, ^3^*J*_HH_= 8,2); 7.98 (m, 1H, -CH=) ppm.

^19^F NMR (CDCl_3_) δ, 2 diastereoisomers, A (50%, rel.), B (50% rel.): -79.7 (m, 2F, O(CF_3_)C*F*_2_); -80.4 (m, 3F, C*F*_3_CF_2_CF_2_); -81.8 (m, 3F, OCF(C*F*_3_)CF_2_); -82.4 (m, 3F, OCF(C*F*_3_)COO); –83.4 (m, 2F, CF_3_CF_2_C*F*_2_); -130.0 (s, 2F, CF_3_C*F*_2_CF_2_-); -132.9 (dd, 1F (A), OC*F*(CF_3_)COO, ^4^*J*_FF_= 20,3 Hz); -133.1 (m, 1F (B), OC*F*(CF_3_)COO); -145.1 (t, 1F (A), OC*F*(CF_3_)CF_2_, ^3^*J*_FF_= 20,9 Hz); -145.5 (m, 1F (B), OC*F*(CF_3_)CF_2_) ppm.

MS (*M_r_*= 801.46 ), *m/z*/ (% rel.int): EI: A: (306 (M^+^-C_3_F_7_CF(CF_3_)CF_2_OCF(CF_3_)CO_2_, 19), 262 (CF_3_CFO^+^CF_2_COCF_3_, 8), 234 (CF_3_CF_2_O^+^CF_2_CF_3_, 5), 214 (4), 180 (3), 169 (C_3_F_7_^+^, 13), 119 (3), 91 (100), 69 (CF_3_^+^, 16), 41 (5), B (306 (M^+^-C_3_F_7_CF(CF_3_)CF_2_OCF(CF_3_)COO, 29), 262 (CF_3_CFO^+^CF_2_C(O)CF_3_, 11), 234 (CF_3_CF_2_O^+^CF_2_CF_3_, 6), 214 (8), 180 (3), 169 (C_3_F_7_^+^, 15), 146 (6), 119 (4), 91 (100), 69 (CF_3_^+^, 15), 41 (5).

**[1-Benzyl-3-butylquinoline-2,4(1*H*,3*H*)-dione-3-yl]-trifluoroacetate (6a):**

Very unstable compound, decomposed on column chromatography, applied in ca. 85% purity.

^19^F NMR (CDCl_3_) δ: -75.10 (s) ppm.

MS (*M_r_*= 419.3 ), m/z/ (% rel.int): 419 (M^+^, 4), 305 (13), 234 (4), 214 (5), 180 (4), 91 (100), 65 (9).

**[1-Methyl-3-phenylquinoline-2,4(1*H*,3*H*)-dione-3-yl]-trifluoroacetate (6b):**

Very unstable compound, decomposed on column chromatography, applied in ca. 80% purity.

^19^F NMR (CDCl_3_) δ: -75.17 (s) ppm.

MS (*M_r_*= 363.3 ), m/z/ (% rel.int): FAB: 364 (M+H^+^, 22).

**[1-Benzyl-3-butylquinoline-2,4(1*H*,3*H*)-dione-3-yl]-acetate (7a):**

The crude product was crystallized (methanol) to afford **7a** as white crystals, yield 160 mg (71 %), m. p 122-125°C.

^1^H NMR (CDCl_3_) δ: 0.86 (t, 3H, C*H*_3_CH_2_ ,^3^*J*_HH_= 7.1 Hz); 1.18-1.52 (m, 4H, CH_2_C*H*_2_C*H*_2_); 1.91-2.17 (ddd, 2H, C*H*_2_-C-OH, ^2^*J*_HH_= 19.8 Hz, ^3^*J*_HH_= 8.3 Hz, ^2^*J*_HH_= 7.2 Hz), 2.25 (s, 3H, CH_3_COO); 5.22 (d,1H, PhC*H*_a_H_b_, ^2^*J*_HaHb_=16.5 Hz), 5.38 (d, 1H, PhCH_a_*H*_b_. ^2^*J*_HbHa_=15.9); 7.03 ( d, 1H, -CH=, ^3^*J*_HH_=8.8 Hz); 7.13 t, 1H, -CH=, ^3^*J*_HH_=7.5 Hz); 7.20-7.38 (m, 5H, -CH=); 7.47 (t, 1 H, -CH=, ^3^*J*_HH_=7.7); 8.01 (d, 1H,-CH=, ^3^*J*_HH_= 7.7 Hz) ppm.

^13^C NMR (CDCl_3_) δ: 14.3 (s, *C*H_3_CH_2_); 20.9 (s, *C*H_3_COO); 23.1 (s, CH_3_*C*H_2_); 25.3 (s, CH_2_*C*H_2_CH_2_); 36.7 (s, *C*H_2_-C-OH); 46.9 (s, Ph*C*H_2_); 84.6 (s, O-*C*-Bu); 116.8 124.1 127.0 128.2 129.3 129.7 136.9 (7x s, -CH=); 121.2 136.4 142.6 (3x s, -C=); 170.5 (s, COO); 171.2 (s, CON); 191.7 (s, PhCO-) ppm.

IR (CHCl_3_): 3027 w, 2962 w, 2932 w, 2876 w, 1744 m, 1712 s, 1679 s, 1602 m, 1469 s, 1374 m, 1247 m, 700 w.

Analysis **(7a)**: For C_21_H_23_O_4_N required: 72.31 % C, 6.36 % H, 3.83 % N; M, 353.41; found: 71.82 % C, 6.65 % H, 3.41 % N.

**[1-Methyl-3-phenylquinoline-2,4(1*H*,3*H*)-dione-3-yl]-acetate (7b):**

The crude product was crystallized (benzene-methanol) to afford ester **7b** as white crystals, yield 947 mg (82 %), m. p. 149-152 °C.

^1^H NMR (CDCl_3_) δ: 2.30 (s, 3H, CH_3_-C); 3.57 (s, 3H, CH_3_-N); 7.18 (t, 1H, -CH= arom., ^3^*J*_HH_=7,4 Hz); 7.20 (d, 1H, -CH= arom., ^3^*J*_HH_=8,8 Hz); 7.28-7.49 (s, 5H, -CH= arom.); 7.64 (t, 1H, -CH= arom., ^3^*J*_HH_=8 Hz); 7.99 (d, 1H, -CH= arom., ^3^*J*_HH_=7,7 Hz) ppm.

^13^C NMR (CDCl_3_): 21.0 (s, CH_3_-C); 30.8 (s, CH_3_-N); 85.6 (s, -*C*-OCOCH_3_); 116.0 121.3 133,2 (3x s, -C= arom.); 124.3 127.4 129.6 129.8 130.6 137.3 143.4 (7x s, -CH= arom.); 169,0 (s, CH_3_-*C*O-O), 171,4 (s, -CO-N), 190, (s, PhCO-) ppm.

IR (CHCl)_3_: 3025 w, 1742 m, 1715 s,1679 s, 1605 m, 1474 s, 1359 m,1247 m, 685 w.

Analysis **(7b)**: For C_18_H_15_O_4_N required: 69.81 % C, 4.89 % H, 4.53 % N; M, 309.32; found: 69.63 % C, 4.94 % H, 4.23 % N.
